# Sex Differences in the Beneficial Cardiac Effects of Chronic Treatment with Atrial Natriuretic Peptide In Spontaneously Hypertensive Rats

**DOI:** 10.1371/journal.pone.0071992

**Published:** 2013-08-12

**Authors:** Mariana Romero, Carolina Caniffi, Gonzalo Bouchet, Rosana Elesgaray, Myriam Mac Laughlin, Analía Tomat, Cristina Arranz, Maria A. Costa

**Affiliations:** Cátedra De Fisiología, Facultad De Farmacia Y Bioquímica, Universidad De Buenos Aires, Instituto De Química Y Metabolismo Del Fármaco, Consejo Nacional De Investigaciones Científicas Y Técnicas, Buenos Aires, Argentina; University of Valencia, Spain

## Abstract

**Introduction:**

The aim of this study was to investigate both the effects of chronic treatment with atrial natriuretic peptide (ANP) on systolic blood pressure (SBP), cardiac nitric oxide (NO) system, oxidative stress, hypertrophy, fibrosis and apoptosis in spontaneously hypertensive rats (SHR), and sex-related differences in the response to the treatment.

**Methods:**

10 week-old male and female SHR were infused with ANP (100 ng/hr/rat) or saline (NaCl 0.9%) for 14 days (subcutaneous osmotic pumps). SBP was recorded and nitrites and nitrates excretion (NOx) were determined. After treatment, NO synthase (NOS) activity, eNOS expression, thiobarbituric acid-reactive substances (TBARS) and glutathione concentration were determined in left ventricle, as well as the activity of glutathione peroxidase (GPx), catalase (CAT) and superoxide dismutase (SOD). Morphological studies in left ventricle were performed in slices stained with hematoxylin-eosin or Sirius red to identify collagen as a fibrosis indicator; immunohistochemistry was employed for identification of transforming growth factor beta; and apoptosis was evaluated by Tunel assay.

**Results:**

Female SHR showed lower SBP, higher NO-system activity and less oxidative stress, fibrosis and hypertrophy in left ventricle, as well as higher cardiac NOS activity, eNOS protein content and NOx excretion than male SHR. Although ANP treatment lowered blood pressure and increased NOS activity and eNOS expression in both sexes, cardiac NOS response to ANP was more marked in females. In left ventricle, ANP reduced TBARS and increased glutathione concentration and activity of CAT and SOD enzymes in both sexes, as well as GPx activity in males. ANP decreased fibrosis and apoptosis in hearts from male and female SHR but females showed less end-organ damage in heart. Chronic ANP treatment would ameliorate hypertension and end-organ damage in heart by reducing oxidative stress, increasing NO-system activity, and diminishing fibrosis and hypertrophy.

## Introduction

It is now universally accepted that blood pressure and the risk of cardiovascular disease is much higher in men than in age-matched premenopausal women, but this difference diminishes after menopause suggesting a protective role of estrogens in the regulation of blood pressure and in cardiovascular and renal protection.

The spontaneously hypertensive rat (SHR) is a model of androgen and angiotensin II-dependent hypertension [1]. SHR present endothelial dysfunction, an increase in oxidative stress and vasoconstrictor factors, a decrease in the bioavailability and effectiveness of nitric oxide (NO), and elevated plasma levels of atrial natriuretic peptide (ANP) [2], [3]. As in humans, SHR exhibit sex differences in blood pressure, with males having higher blood pressure than females [4].

On the other hand, essential hypertension often leads to hypertrophic cardiomyopathy [5]. In this regard, SHR develops left ventricular hypertrophy, showing an increased cardiac fibrosis, in response to elevated ventricular volume or pressure overload [6].

It is well known that estrogens affect cardiovascular function, either by decreasing blood pressure directly or by modifying production of endothelium-derived factors [7]–[9]. In ovariectomized rats, estradiol treatment reduces blood pressure and increases the synthesis and release of ANP [10]. Indeed, in human studies, higher circulating levels of ANP have been observed in premenopausal women, compared with men [11], [12]. http://atvb.ahajournals.org/cgi/content/full/26/7/1524-R11-099283#R11-099283 http://atvb.ahajournals.org/cgi/content/full/26/7/1524-R12-099283#R12-099283 http://atvb.ahajournals.org/cgi/content/full/26/7/1524-R13-099283#R13-099283 We have previously provided evidence that acute ANP injection induces a hypotensive effect by enhancement of cardiovascular NO-synthase (NOS) activity in both normotensive and hypertensive rats [13]–[15]. In addition, it has been shown that the anti-inflammatory, anti-apoptotic and anti-fibrotic effects of estrogens on the cardiovascular system could be mediated, at least in part, by NO production [16].

Oxidative stress is an imbalance between the production of free radicals and antioxidant defense mechanisms. Increased production of reactive oxygen species (ROS) has been associated with development of hypertension [17]. ANP exerts protective effects against oxidative stress and in human studies it has been shown that ANP infusion has antioxidant effects, reducing superoxide anion production in myocytes [18]. On the other hand, the inhibition of phospholipase D activity, as well as the decrease of pH and intracellular calcium by ANP, is a way of protecting the vascular wall against oxidative stress [19]. In addition, ANP activates cardiomyocytes particulate guanylyl cyclase-coupled natriuretic peptide receptor A (NPR-A) and increases intracellular cGMP levels. The subsequent cGMP stimulation of protein kinase G suppresses the induction of NADPH oxidase, and hence decreases the amount of superoxide generated by this enzyme [20]. Experimental studies show that genetic deletion of estrogen receptor β results in hypertension in male and female mice [21] and in hypertensive rats, and estrogen deficiency results in endothelial dysfunction and oxidative stress [22].

We therefore hypothesized that chronic ANP treatment in SHR would ameliorate hypertension and end-organ damage in heart by reducing oxidative stress, increasing NO-system activity, and diminishing fibrosis and hypertrophy. Moreover, as a relationship between ANP and estrogens is thus postulated, the effects of the treatment could be different in male and female SHR.

In order to demonstrate this hypothesis, we investigated the effects of chronic treatment with ANP on systolic blood pressure, cardiac NO system, oxidative stress, hypertrophy, fibrosis and apoptosis in SHR. Moreover, this study also looked into possible sex-related differences in the response to the treatment.

## Materials and Methods

### Animals

Ten-week old male and female SHR were purchased from the Instituto de Investigaciones Médicas A. Lanari, Facultad de Medicina (Universidad de Buenos Aires, Argentina). Rats were housed in a humidity and temperature-controlled environment with an automatic 12-hour light-dark cycle. They were fed standard rat chow from Nutrimentos Purina (Buenos Aires, Argentina) and provided tap water *ad libitum* up to the day of the experiments.

### Experimental design

All experimental protocols were performed in accordance with the Guide for the Care and Use of Laboratory Animals (National Institutes of Health, Publication No. 85-23, Revised 1996) and Regulation No. 6344/96 of Argentina's National Drug, Food and Medical Technology Administration (ANMAT). Experimental procedures were approved by the Ethics Committee of the School of Biochemistry and Pharmacy, Universidad de Buenos Aires.

### Protocol

Animals were separated by sex and then randomly assigned to the ANP-treated group (n = 10): chronic infusion with ANP (100 ng/h/rat), or the Control group (n = 10): chronic infusion with NaCl 0.9%, for 14 days. Chronic infusion in both groups was performed using an Alzet micro-osmotic pump (model 1002), prepared according to the manufacturer's instructions, and implanted subcutaneously between the scapulae under light ether anesthesia using aseptic technique.

Systolic blood pressure (SBP) was recorded and urine samples were collected at the end of the experimental period in all groups of animals. SBP was measured in awake animals (tail cuff method) with a MP100 Pulse Transducer, PanLab (Quad Bridge Amp, ADInstruments), and recorded with a polygraph (Quad Bridge Amp, ADInstruments). Data were obtained using data acquisition software (PowerLab 8/30 and Labchart, Australia).

The concentration of nitrites and nitrates (NOx), end products derived from NO metabolism, was determined in urine samples collected over 24 hours according to the procedure described by Verdon et al. [23].

Subsequently, animals were sacrificed by decapitation and hearts were removed and weighed in order to evaluate NOS activity and expression, oxidative stress, fibrosis and apoptosis.

### Determination of NOS activity

NOS activity in the left ventricle (LV) was measured using [^14^C] L-arginine as substrate, as described previously [24], [25]. 2–3 mm thick tissue slices were incubated 30 minutes at 37°C in Krebs solution with 0.5 µCi/ml [^14^C] L-arginine. The reaction was stopped by adding 500 µl stop buffer containing 0.5 mM EGTA, 0.5 mM EDTA and 20 mM HEPES (pH 5.5). Tissue samples were then homogenized in the stop solution and the homogenates were centrifuged at 12,000 g for 20 minutes.

The supernatants were then applied to a 1 ml Dowex AG 50W-X8 column (Na^+^ form, Bio-Rad), hydrated with the stop buffer, and eluted with 2 ml distilled water. The amount of [^14^C] L-citrulline was determined with a liquid scintillation counter (Wallac 1414 WinSpectral). Specific NOS activity was assessed in the presence of 10^−4^ M L-Nitro arginine methyl ester (L-NAME, Sigma). NO production in each tube was normalized to the weight of the tissue slices incubated with the substrate for equal periods of time and expressed as picomoles of [^14^C] L-citrulline per gram wet weight per minute.

### Western blot analysis

Samples of LV tissue containing equal amounts of protein (0.10 mg protein/lane) were separated by electrophoresis in 7.5% SDS-polyacrylamide gels, transferred to a nitrocellulose membrane (Amersham G.E. Healthcare), and then incubated with rabbit polyclonal anti-NOS antibodies (1/500 dilution) and a horseradish peroxidase-conjugated goat anti-rabbit secondary antibody (1/5,000 dilution: anti-eNOS, epitope at the NH_2_ terminus) (Santa Cruz Biotechnology, Santa Cruz, CA). A marker of β-actin was used as a loading control and data were normalized to β-actin expression. Samples were revealed by chemiluminescence using an enhanced chemiluminescence reagent (Amersham Pharmacia Biotechnology, Uppsala, Sweden) for 2–4 minutes. Quantification of the bands was performed by digital image analysis using a Hewlett-Packard scanner and Totallab analyzer software (Biodynamics, Seattle, WA). All experiments were performed in triplicate.

### Oxidative stress evaluation

LV slices were homogenized (OMNI MIXER homogenizer) in 30 mM phosphate buffer potassium, pH 7.4, 120 mM KCl (1 g tissue/10 ml buffer), and centrifuged at 2,500 rpm for 10 minutes at 4°C. Lipid oxidative damage was assessed by measuring the extent of formation of 2-thiobarbituric acid reactive substances (TBARS; nmol/mg protein) [26]. Super oxide dismutase (SOD) activity was assessed by measuring the ability of the homogenate to inhibit autoxidation of epinephrine, and was expressed as units of SOD per milligram of protein [27]. Catalase (CAT) activity was determined by the conversion of hydrogen peroxide to oxygen and water, and was expressed as picomole per milligram of protein [28]. The assay described by Flohé and Gunzler was used to measure glutathione peroxidase (GPX) activity, and was expressed as nanomoles per minute per milligram of protein [29].

In order to measure glutathione content, LV slices were homogenized in 100 mM phosphate buffer sodium, pH 7.0, 5 mM EDTA (1.6 g tissue/10 ml buffer), and centrifuged at 13,000 rpm for 20 minutes at 4°C. Glutathione concentration was measured according to the method described by Tietze F. and was expressed as milligram per milligram of protein [30]. Protein concentration was determined by the method of Bradford et al. [31].

### Histological evaluation and immunolabeling

LV was cut longitudinally, fixed in phosphate-buffered 10% formaldehyde, pH 7.2, and embedded in paraffin wax. Tissue sections (3 µm) were stained with hematoxylin and eosin. Ventricle morphometric parameters were determined in 10 areas, measuring major and minor diameters and then calculating mean diameter.

Ventricle sections were stained with the collagen-specific stain Picrosirius Red to determine the presence of fibrosis, as described previously [32]. Collagen staining was evaluated and scored: 0 =  normal and slight staining surrounding vascular structures; 1 =  (mild) weak staining that doubles normal label surrounding vascular structures; 2 =  (moderate) moderate staining in the interstitium surrounding cardiomyocytes; 3 =  (severe) strong staining in the interstitium surrounding cardiomyocytes and compromising <25% of the area; and 4 =  (very severe) strong staining in the interstitium surrounding cardiomyocytes and compromising >25% of the area. A score was assigned to each section, mainly reflecting the changes in extent rather than in intensity of staining.

Immunohistochemistry for transforming growth factor beta (TGF-β) was performed on formalin-fixed, paraffin-embedded samples sectioned at 5 µm. Serial sections were deparaffinized in xylene and rehydrated through a graded series of ethyl alcohol and PBS. Endogenous peroxide was blocked by incubation in peroxidase blocking reagent DAB (Dako EnVision ® + system-HRP) for 5 minutes. After washing with PBS, sections were incubated for 40 minutes with primary polyclonal antibody against Smad protein (H465 sc 7153, dilution 1∶100) (Santa Cruz Biotechnology, Santa Cruz, CA). After washing, sections were incubated with the secondary antibody. Sections were developed with 3,3′-diaminobenzidine solution as chromogen for 15 minutes, counterstains with hematoxylin, dehydrated, cleared and mounted. Negative controls were performed by omitting the primary antibodies. Results are expressed as the percentage of the total area that presents staining (% stained area/total area).

### Tunel

The DeadEnd Colorimetric TUNEL System, a non-radioactive kit designed to end-label the fragmented DNA of apoptotic cells, was used as previously described [30]. The number of TUNEL-positive cells per cardiac area was counted in 20 visual fields (magnification X400) for each rat.

Histological, immunohistological and TUNEL assays were analyzed using an Olympus BX51 light microscope equipped with a digital camera (Qcolor 3, Olympus America, Inc., Richmond Hill, Ontario, Canada) and connected to the Image-Pro Plus 4.5.1.29 software (Media Cybernetics, LP, Silver Spring, MD, USA). The measurements were performed blindly and under similar light, gain, offset, and magnification conditions.

### Statistical Analysis

All values are expressed as means ±SEM. The Prism program (Graph Pad Software, Inc., San Diego, CA, USA) was used for statistical analysis. The mean and standard error of median values of each variable were calculated for each group. The results of each variable for each experimental group were analyzed with a two-way analysis of variance (ANOVA), where one factor was the different treatments and the other was sex (male or female). The effects of one factor were tested independently of the effects of the other, as well as the interaction between both factors. No interaction between treatments and genotype was found. Multiple comparisons were performed using a Bonferroni post hoc test. p value <0.01 was considered a significant difference.

## Results

### Effects of chronic treatment with ANP on SBP and the NO-system

**Figure 1:**
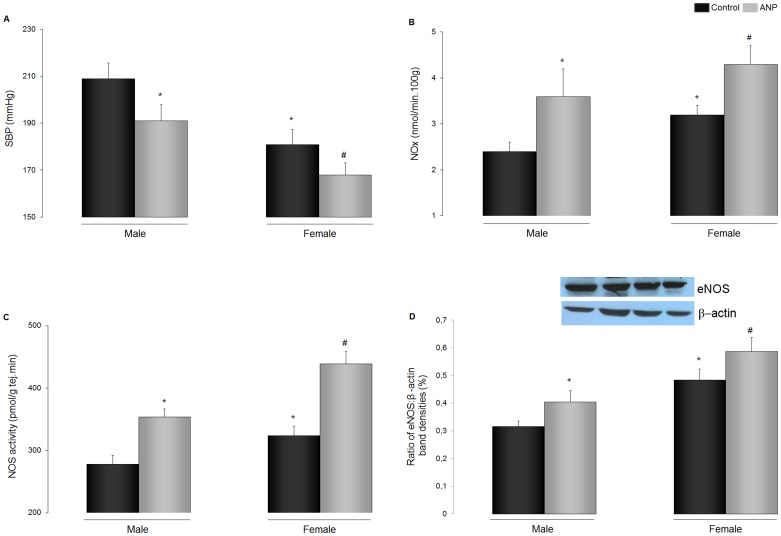
ANP treatment effects on SBP, NOx, cardiac NOS activity and eNOS expression. (**A**) SBP: systolic blood pressure; (**B**) NOx: Nitrites and nitrates excretion; (**C**) NOS (nitric oxide synthase) activity; (**D**) Representative blot of eNOS and β-actin and quantification of the bands of eNOS. Data are mean ± SEM (n = 10). *p<0.01 vs. Control male, # p<0.01 vs Control female.

Figure 1A shows SBP in control and ANP-treated male and female SHR. Male SHR showed higher levels of SBP than females. Chronic treatment with ANP lowered SBP in both sexes (Figure 1A).

NO systemic production was evaluated by measurement of NOx excretion, and basal NOx excretion was found to be higher in female than in male SHR. ANP chronic infusion increased NOx excretion in both sexes (Figure 1B).

To verify whether this increase in NOx was associated with an increase in cardiac NOS activity and/or expression, and whether any sex differences existed, NOS activity and eNOS expression were measured in cardiac ventricle. The activity of the enzyme was higher in female than in male SHR, NOS activity increased significantly in both sexes after ANP treatment but the response of cardiac NOS to ANP treatment was more marked in female SHR (Figure 1C). eNOS protein content was higher in female than in male ventricles, and ANP increased eNOS expression in both sexes (Figure 1D).

### Study of heart oxidative stress in response to chronic ANP treatment

In the control group female rats displayed higher levels of glutathione and lower levels of TBARS when compared to males. Chronic treatment with ANP increased the levels of glutathione and reduced TBARS in both sexes. The anti-oxidant enzymes that participate in the regulation of ROS were then studied and female rats showed higher basal activity of CAT and SOD, but reduced GPx activity, compared to males. In addition, chronic treatment with ANP increased CAT and SOD activity only in male SHR, while ANP treatment decreased GPx activity in both sexes (Table 1).

**Table 1:** Effects of chronic treatment with ANP on oxidative stress in left ventricle of male and female SHR.

	Male		Female	
	Control	ANP	Control	ANP
GSH (mg/mg tissue)	0.183±0.032	0.268±0.047*	0.365±0.061*	0.424±0.054#
TBARS (nmol/mg prot)	0.318±0.036	0.218±0.023*	0.150±0.019*	0.114±0.017#
CAT (pmol/mg prot)	0.184±0.042	0.452±0.079*	0.559±0.084*	0.513±0.071
SOD (USOD/mg prot)	11.89±1.01	15.22±1.09*	15.45±1.27*	16.10±1.51
GPx (µmol/min.mg prot)	332.1±26.4	243.2±19.2*	239.4±18.1*	198.3±11.7#

GSH: glutathione; TBARS: thiobarbituric acid reactive substances; CAT: Catalase; SOD: superoxide dismutase; GPx: gluthatione peroxidase.

Data are mean ±SEM (n = 10).

*p<0.01 vs. Control male;

# p<0.01 vs. Control female.

### Changes in body and heart dimensions after treatment with ANP

Body, heart and LV weight were higher in male than in female rats. In this regard, a significant decrease in these parameters was noted in male rats after chronic treatment with ANP, but only heart weight was reduced in treated female rats. The LV/BW ratio, as a parameter of LV hypertrophy, was higher in male rats, and it was reduced after ANP treatment (Table 2).

**Table 2:** Body weight and heart dimensions in control and ANP-treated male and female SHR.

	Male		Female	
	Control	ANP	Control	ANP
BW (g)	309.6±6.31	273.5±8.72*	206.8±4.21*	208.7±3.15
HW/BW (mg/g)	0.482±0.020	0.436±0.018*	0.463±0.021*	0.421±0.017#
LV (g)	1.186±0.015	0.894±0.099*	0.701±0.023*	0.665±0.013
LV/BW (mg/g)	0.38±0.01	0.33±0.01*	0.34±0.01*	0.31±0.01
mCD (µm)	27.81±0.34	25.01±0.46*	24.09±0.69*	20.71±0.89#

BW: body weight; HW: heart weight; LV: left ventricle; mCD: mean cardiomyocyte diameter.

Data are mean ±SEM (n = 10).

*p<0.01 vs. Control male;

# p<0.01 vs. Control female.

Mean myocyte diameter was measured in LV slices and female rats showed reduced myocyte size compared with males. ANP treatment reduced mean cardiomyocyte diameter in both sexes (Table 2).

### Sirius Red staining, TGF-β immunohistochemistry and apoptosis in cardiac ventricle

**Figure 2:**
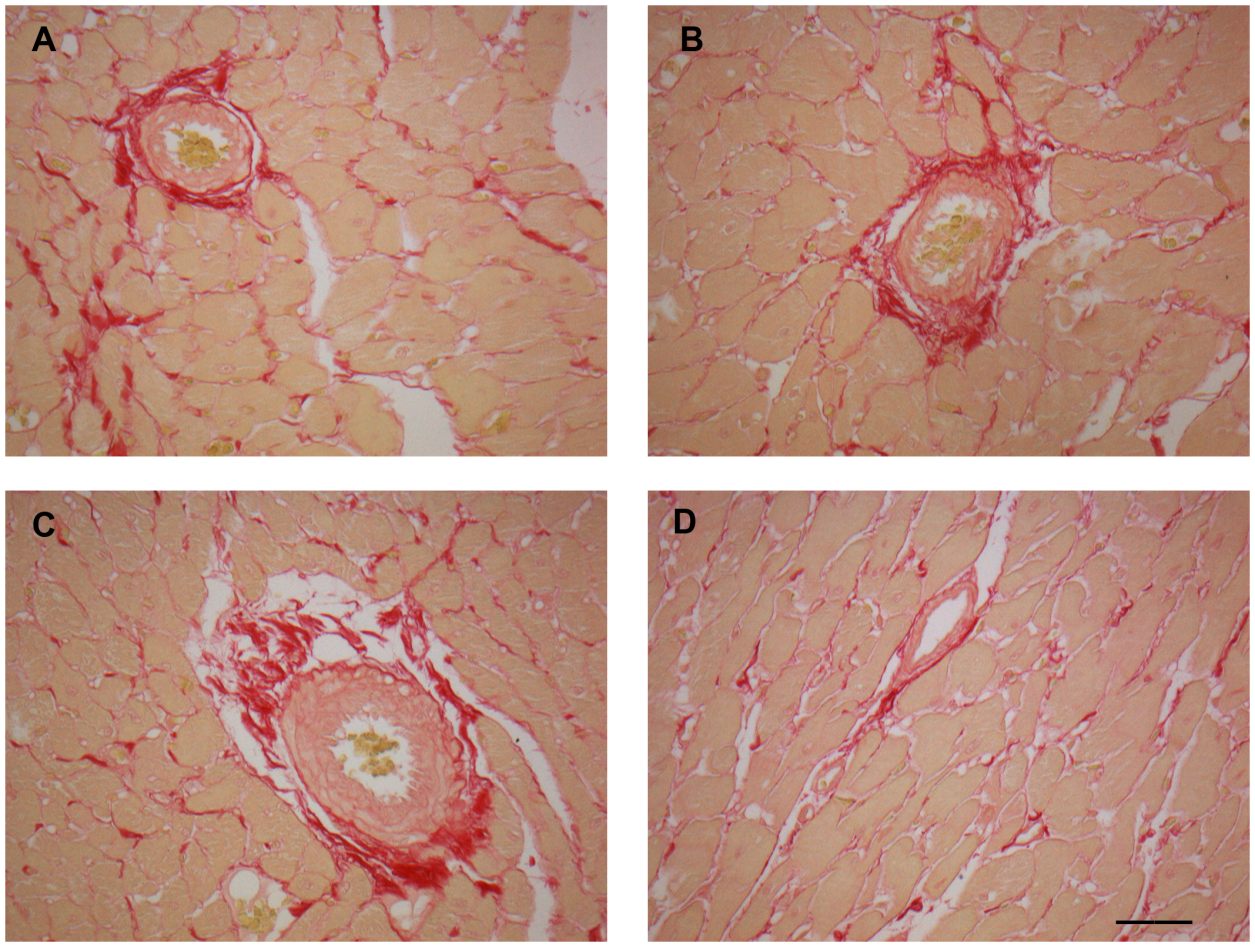
Sirius Red staining in left ventricle after treatment with ANP. The red colour of Sirius red staining under the common microscope indicates total collagen deposits, representative images are from hearts of each group. (**A**): Control male; (**B**): ANP male; (**C**): Control female; (**D**): ANP female. All images are in the same magnification 400X. Scale bar  = 30 µm.

**Figure 3:**
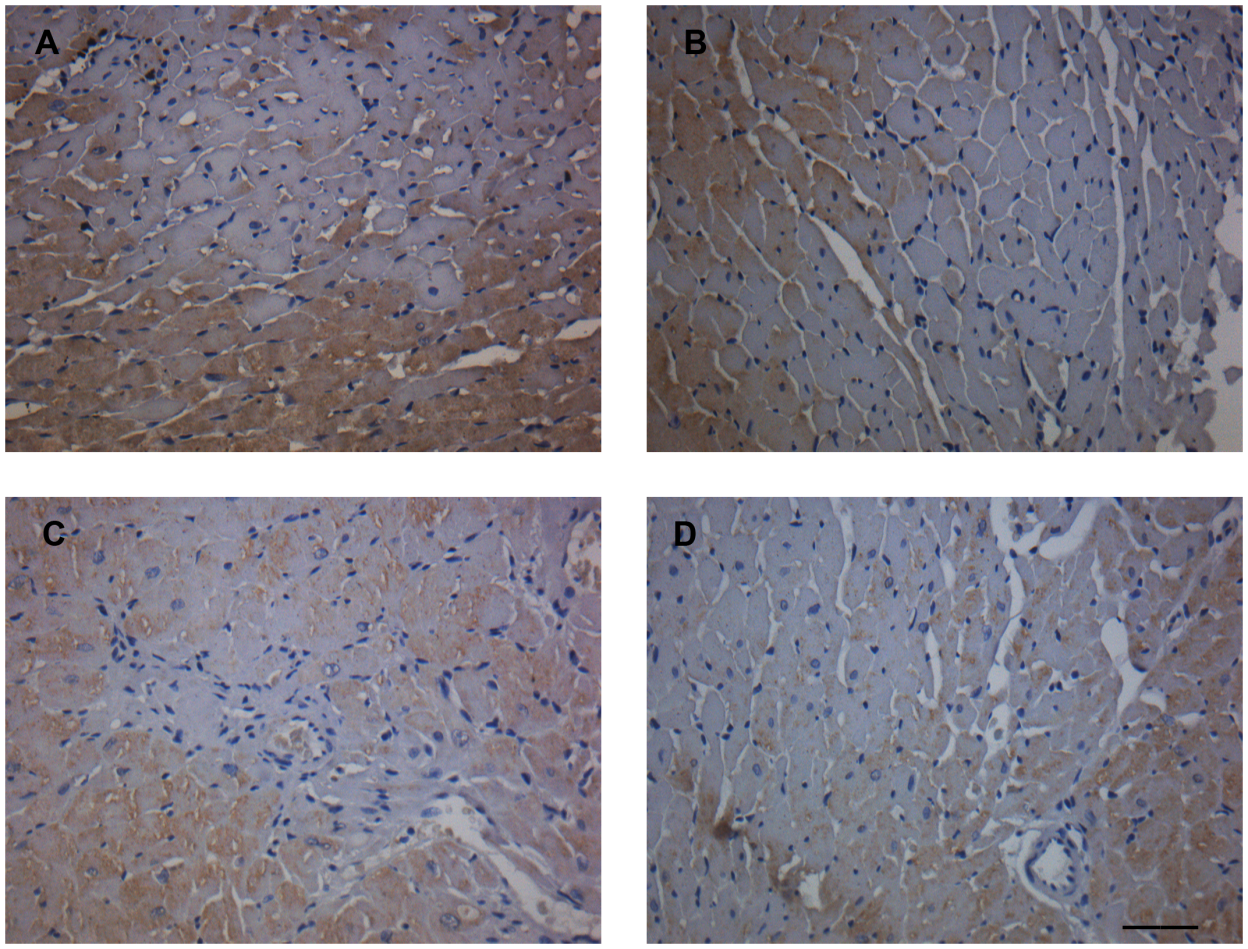
Immunohistochemistry staining for TGF-β in left ventricle after treatment with ANP. Representative micrographs of immunostained TGF-β in the hearts from each group. (**A**): Control male; (**B**): ANP male; (**C**): Control female; (**D**): ANP female. All images are in the same magnification 400X. Scale bar  = 30 µm.

LV sections stained with collagen-specific Picrosirius Red showed that female rats had less fibrosis than male rats and ANP treatment reduced the staining for collagen type 1 and 3 in both sexes (Table 3, Figure 2). Immunohistochemistry for TGF-β as a factor responsible for LV fibrosis in hypertension was also evaluated, and the same behavior was found: female rats showed a lower percentage of staining for this factor than male rats. ANP treatment reduced staining for TGF-β in both sexes (Table 3, Figure 3).

**Table 3:** Effects of chronic treatment with ANP on cardiac fibrosis and apoptosis in male and female SHR.

	Male		Female	
	Control	ANP	Control	ANP
SR (Score)	2.30±0.09	2.03±0.10*	2.02±0.12*	1.75±0.10#
TGF-β (%)	18.11±1.26	11.51±1.16*	14.91±1.47*	11.41±0.96#
Apoptotic/total cells (%)	11.61±0.94	5.97±0.38*	9.07±1.32	5.16±0.47#

SR: Sirius red; TGF-β: transforming growth factor beta.

Data are mean ±SEM (n = 10).

*p<0.01 vs. Control male;

# p<0.01 vs. Control female.

Examination of TUNEL-stained LV sections of control rats revealed no differences between males and females in the number of apoptotic cells, but treatment with ANP decreased the number of apoptotic cells in both male and female SHR (Table 3).

## Discussion

This is the first experimental in vivo study that shows that chronic treatment with ANP reduces cardiac oxidative stress, fibrosis, apoptosis and hypertrophy in a model of hypertension, increasing NO-system activity. A sex-related difference in the cardiac response to ANP in SHR is also described.

In previous studies we demonstrated that male SHR showed increased urinary excretion of NO metabolites and augmented activity of cardiovascular and renal NOS compared to normotensive animals, indicating that the NO pathway is up-regulated in this model of hypertension [15], [33]. In the present study, we showed that female SHR exhibit lower SBP than male SHR. Female SHR also presented an increase in NOx, higher activity and expression of cardiac eNOS and a reduction in LV oxidative stress compared to male rats, indicating a sex difference in these parameters. Our results are consistent with different clinical and experimental studies that demonstrate sex differences in the prevalence of hypertension before the onset of menopause. This fact could be attributed to the beneficial effects of estradiol on the cardiovascular system, which include vasodilatation by a direct mechanism and, indirectly, by increasing eNOS activity in blood vessels [6], [34]–[37]. Furthermore, it has been shown in humans and animals that NO level is higher in females than in males because estrogens not only stimulate NO production but also decrease inactivation of NO by oxygen radicals [8], [9], [38]. Moreover, epidemiological and experimental evidence suggests that oxidative stress is enhanced in males compared with females [39]–[41]. Our results show that hearts of female SHR exhibit higher levels of glutathione and lower content of TBARS than male ones, indicating a sex difference in cardiac oxidative stress. In addition, cardiac activity of CAT and SOD was higher in female than in male SHR, suggesting a more effective antioxidant system in heart of female SHR. Moreover, higher GPx expression in the heart of male rats compared with females [42] has been reported. In this regard, we found increased GPx activity in male SHR compared with female SHR, probably due to a compensatory response to the lower levels of glutathione observed in male SHR.

Several studies have shown increased oxidative stress associated with higher levels of hypertrophy in hearts from male SHR in comparison to Wistar rats [43]. This study showed that female SHR presented less LV fibrosis and a lower index of LV hypertrophy than male SHR. Moreover, mean diameter of cardiac myocytes was smaller in female than in male rats. We found that, in this model of hypertension, female rats showed not only less cardiac fibrosis but also less profibrotic factor TGF-β than male rats. These results are supported by in vitro findings which demonstrate that estrogen inhibits the hypertrophic response and exhibits an antifibrotic effect on the heart by inhibiting transition from fibroblast to myofibroblast, diminishing the synthesis of collagen type 1 and 3 and suppressing the profibrotic agent TGF-β [44].

It has been reported that SHR show higher levels of apoptosis than WKY [45]. Pelzer T. et al. demonstrated that estradiol inhibits apoptosis induced by oxidative stress in cardiac myocytes [46]. In this regard, our results showed that while females exhibit lower oxidative stress in LV than males, we found no sex differences in cardiac apoptosis in SHR. On the other hand, it is well known that ANP is a diuretic, natriuretic and hypotensive factor. In previous studies, we found that acute treatment with ANP in normotensive and hypertensive male animals reduces blood pressure, increasing cardiovascular NOS activity by interacting with natriuretic peptide receptors NPR-A and NPR-C [33]. Therefore, we tested the hypothesis that long-term treatment with ANP induces cardiovascular benefits in SHR and sex differences are present in this model of hypertension. Consistent with our hypothesis, we found that this treatment lowered SBP in both sexes, and it was accompanied by an increase in NO-system activity and a decrease in oxidative stress in the heart. The response of cardiac NOS to ANP in female rats was higher than in males, showing a probable role of estrogen in enhancement of ANP effects on the NO-system.

Natriuretic peptides were found to attenuate ROS production by interacting with all three receptor classes. Thus, in hepatocytes and Kupffer cells, ANP reduces oxidative stress by activation of NPR-A/B [47]. In turn, enhanced oxidative stress in vascular smooth muscle cells of SHR was reduced by activation of NPR-C with its specific agonist, cANP (4–23) [48]. It has been recently shown that the infusion of human synthetic ANP (carperitide) in patients with heart failure not only afforded beneficial effects on hemodynamic performance but also acted as an antioxidant [49], [50]. In addition, in neonatal rat cardiomyocytes cultured with angiotensin II, several hypertrophic responses such as increase in cardiomyocyte size and superoxide generation were reduced in the presence of ANP [20]. Our study supports these findings and shows that chronic treatment with ANP reduces cardiac oxidative stress in both male and female SHR, probably due to a decrease in superoxide production and an increase in NO synthesis. In addition, the treatment with ANP also increased CAT and SOD activity in male hearts, contributing to reduce oxidative stress, which was more marked in male than in female rats.

With respect to the antihypertrophic properties of ANP, Oliver et al. reported that NPR-A deficiency in mice leads to cardiac hypertrophy, much higher fibrosis and elevated blood pressure [51]. In the present study, when SHR were treated with a chronic infusion of ANP, the index of LV hypertrophy was reduced only in male rats, which also exhibited a reduction in mean diameter of myocytes. ANP presents an antifibrotic effect in sexes, reducing the percentage of collagen type 1 and 3 and diminishing TGF-β in LV.

In vitro data about the role of ANP in regulation of apoptotic mechanisms appears controversial. Studies performed by Wu C. et al. in neonatal rat cardiac myocytes showed that ANP induces apoptosis, inhibiting de expression of Mcl-1, which is an anti-apoptotic homolog of Bcls [52]. In contrast, Kato et al. observed that ANP promotes cardiomyocyte survival by nuclear accumulation of Akt and zyxin (in a cGMP-dependent mechanism) [53]. The present protocol represents the first in vivo study that analyzes the effects of ANP treatment on apoptosis in the heart, showing that chronic treatment with the peptide reduces apoptosis in the cardiac ventricle of both male and female SHR.

It is well known that NO exerts antioxidant, antifibrotic and antihypertrophic effects in the heart [54], [55]. Additionally, in the present study we demonstrated that ANP increases cardiac eNOS activity and expression. Based on these findings, we can postulate that indirect effects of ANP in LV would be mediated, at least in part, by the increase in NO, which also contributes to reduce oxidative stress.

Whether sex plays an essential role in the onset of hypertension complications and organ damage continues to be an issue of intense debate, but we found that, in this model of hypertension, female SHR showed less cardiac fibrosis, apoptosis and oxidative stress in the heart than male SHR. While we must take into account that males have higher blood pressure values than females, it is important to consider that blood pressure values in females are also consistent with target organ damage. On the other hand, chronic treatment with ANP not only lowered SBP but also induced antifibrotic, antihypertrophic and antiapoptotic effects on the heart of male and female SHR, showing more benefits in males which present major organ damage. In accordance with our results, the beneficial effects of ANP in hypertension probably involve activation of the NO-system and reduction in ROS levels.
